# Clinical outcomes of colonoscopic polypectomy with strategic surveillance colonoscopies in patients with 10 or more polyps

**DOI:** 10.1038/s41598-023-29604-x

**Published:** 2023-02-14

**Authors:** Jin Hwa Park, Seung Wook Hong, Sung Wook Hwang, Sang Hyoung Park, Dong-Hoon Yang, Byong Duk Ye, Seung-Jae Myung, Suk-Kyun Yang, Jeong-Sik Byeon

**Affiliations:** 1 Department of Gastroenterology, University of Hanyang College of Medicine, Seoul, Republic of Korea; 2 Department of Gastroenterology, University of Ulsan College of Medicine, Asan Medical Center, Seoul, Republic of Korea

**Keywords:** Cancer screening, Colonoscopy, Gastrointestinal diseases

## Abstract

The clinical usefulness of repeat colonoscopic polypectomy in patients with numerous polyps has not been sufficiently determined. We aimed to analyze the clinical outcomes of colonoscopic polypectomy with surveillance colonoscopies in patients with ≥ 10 polyps. We reviewed the medical records of 152 patients who underwent polypectomy of ≥ 10 polyps at the baseline colonoscopy. We investigated polyp number, polyp size, polypectomy method, procedure time, and adverse events of the baseline colonoscopy. We also investigated the frequency and interval of surveillance colonoscopies and their findings. The mean number of polyps detected at the baseline colonoscopy was 20.0, of which 16.0 polyps were endoscopically resected. The mean size of the largest polyp was 13.4 mm. The mean procedure time was 54.9 min. Post-polypectomy bleeding occurred in 6 (3.9%) patients, all of whom were treated conservatively. No patients developed perforation. With an increasing number of surveillance colonoscopies, the number of detected polyps and the procedure time decreased. Surveillance colonoscopies identified colorectal cancer only in three patients (2.0%), all of which were mucosal cancers that could be curatively treated by polypectomy. Colonoscopic polypectomy with repeat surveillance colonoscopies is a clinically effective, efficient, and safe management option in patients with ≥ 10 polyps.

## Introduction

Colorectal cancer is one of the most common cancers worldwide and the third leading cause of cancer-related death^1^. Since most colorectal cancers occur through the adenoma-carcinoma sequence, early endoscopic detection and removal of precancerous lesions can lower the incidence of and mortality from colorectal cancer^2,3^. Therefore, repeat regular surveillance colonoscopy after baseline screening colonoscopy is recommended. International guidelines suggest the interval of surveillance colonoscopy should be determined according to the baseline colonoscopy findings, such as the number, size, and histology of detected adenomas^4,5^.

The consensus update by the U.S. Multi-Society Task Force (USMSTF) on Colorectal Cancer in 2020 recommends a 3-year interval for surveillance colonoscopy after endoscopic removal of 5–10 tubular adenomas or ≥ 10 mm because of the increased risk of metachronous advanced neoplasia in patients with multiple adenomas^5^. In addition, the current guideline by the European Society of Gastrointestinal Endoscopy (ESGE) in 2020 also recommends a 3-year interval for surveillance colonoscopy after endoscopic removal of ≥ 5 adenomas^4^. Interestingly, while the ESGE guidelines do not specify the surveillance colonoscopy interval for those with removal of > 10 adenomas, the USMSTF recommends a 1-year interval for surveillance colonoscopy after endoscopic removal of > 10 adenomas. However, the strength of the recommendation was weak, and the quality of evidence was very low, indicating the need for further studies on the surveillance strategy after endoscopic removal of > 10 adenomas.

Endoscopic resection is the standard treatment for colon polyps. Depending on the size, shape, and histological diagnosis, various methods can be used, such as cold snare polypectomy (CSP), endoscopic mucosal resection (EMR), and endoscopic submucosal dissection (ESD). The adverse events include bleeding and perforation; however, these methods are generally considered safe. The incidences of delayed bleeding and perforation after CSP and EMR vary from 0.3 to 7.2% and from 0.08 to 1.3%, respectively. The incidences of ESD-associated delayed bleeding and perforation varies from 1 to 10%, depending on the skill of the endoscopist. Post-polypectomy coagulation syndrome occurs in 1.4–3.7% of patients^6–10^. The risk of adverse polypectomy events increases with the number of polyps. Therefore, caution is required when performing endoscopic resection of many polyps, particularly with repeated resection.

This study investigated the long-term clinical outcomes of repeat endoscopic resection of multiple colorectal polyps in patients with ≥ 10 adenomas on a baseline colonoscopy. In addition, we assessed the effectiveness and safety of repeat endoscopic resection in preventing metachronous advanced neoplasia, suggesting an appropriate surveillance endoscopy strategy for these patients.

## Results

### Baseline characteristics of patients

The mean age of the 152 patients at the time of baseline colonoscopy was 60.9 years, and 122 (80.3%) patients were men. Twenty-two (14.5% patients) had a family history of colorectal cancer. Only 6 (3.9%) patients underwent genetic testing; 5 showed genetic mutations related to hereditary polyposis syndromes (mutation of *APC*, *EXO1*, and *STK11*). Detailed baseline characteristics are presented in Table 1.

**Table 1:** Baseline characteristics of patients.

Characteristics	
Age (year)	60.9 ± 11.2 (median 62.5, range 25–81)
Sex	
Male	122 (80.3%)
Female	30 (19.7%)
Genetic tests performed	
No	146 (96.1%)
Yes	6 (3.9%)
No mutations detected	1 (0.7%)
Relevant mutations detected	5 (3.2%)
* APC* mutation	2 (1.3%)
* EXO1* mutation	1 (0.6%)
* STK11* mutation	2 (1.3%)
Smoking status	
Current smoker	41 (27.0%)
Ex-smoker	33 (21.7%)
Never-smoker	78 (51.3%)
Alcohol consumption	
Yes	81 (53.3%)
No	71 (46.7%)
Body mass index (kg/m^2^)	23.6 ± 3.1 (median 23.1, range 18.6–30.4)
Family history of colorectal cancer	22 (14.5%)

Data are presented as n (%) or mean ± standard deviation with median and range.

*APC* adenomatous polyposis coli, *EXO1* exonuclease 1, *STK11* serine/threonine kinase 11.

### Baseline colonoscopy findings

**Figure 1:**
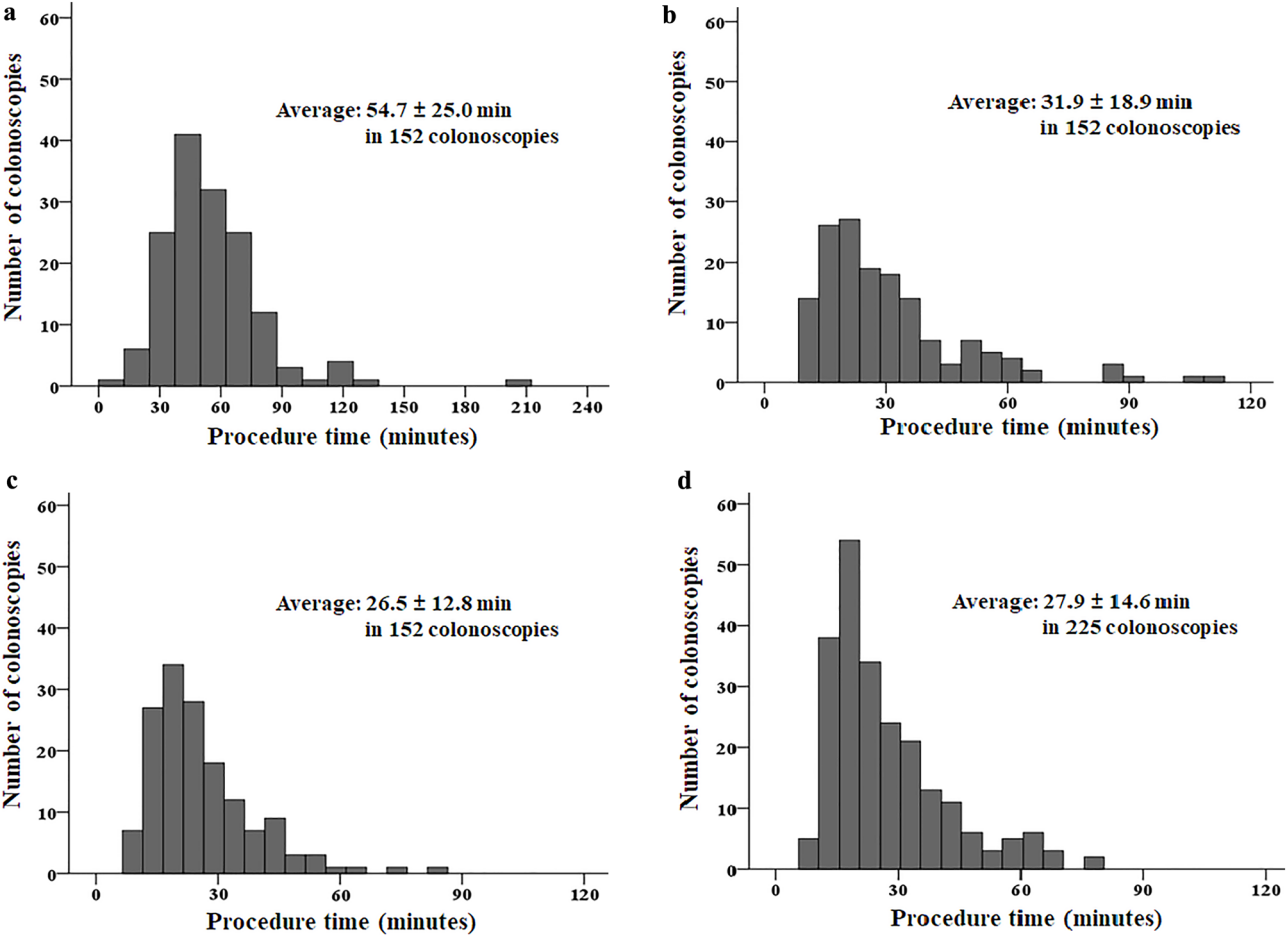
Colonoscopy procedure time (**a**) baseline colonoscopies, (**b**) first surveillance colonoscopies, (**c**) second surveillance colonoscopies, and (**d**) third and subsequent surveillance colonoscopies.

The mean number of polyps detected at baseline colonoscopy was 20.0 ± 22.8 (median 13, range 10–200). According to these, 16.0 ± 12.3 (median 13, range 10–147) were endoscopically resected. The mean size of the largest polyp was 13.4 ± 6.3 mm (median 12.0 mm, range 3.0–40.0 mm). The procedure time ranged from 20 to 210 min (54.9 ± 24.7 min). Only 10 (6.6%) of 152 baseline colonoscopies took > 90 min (Table 2, Fig. 1). EMR was the most frequently performed polypectomy method (1336 polyps, 55.7%), followed by cold forceps polypectomy (CFP) (635 polyps, 26.4%), CSP (411 polyps, 17.1%), endoscopic piecemeal mucosal resection (EPMR) (14 polyps, 0.6%), and ESD (4 polyps, 0.2%). Among the 2370 specimens retrieved and analyzed, 2063 (87.0%) were adenomas, 22 (0.9%) were sessile serrated lesions, 16 (0.7%) were mucosal cancers, and 10 (0.4%) were superficial submucosal cancers with submucosal invasion depth < 1000 µm (Table 2). Delayed bleeding occurred in 6 (3.9%) patients. All cases of delayed bleeding were successfully managed conservatively using endoscopic hemostasis. None of the patients developed perforations.

**Table 2:** Baseline colonoscopy findings.

Characteristics	
Number of polyps detected	20.0 ± 22.8 (median 13, range 10–200)
Number of polyps resected	16.0 ± 12.3 (median 13, range 10–147)
Size of the largest polyp, mm	13.4 ± 6.3 (median 12.0, range 3–40)
Colonoscopy procedure time, min	54.9 ± 24.7 (median 50, range 20–210)
< 30 min	14 (9.2%)
30–59 min	87 (57.2%)
60–89 min	41 (27.0%)
≥ 90 min	10 (6.6%)
Colonoscopic polypectomy methods*	
CFP	635 (26.4%)
CSP	411 (17.1%)
EMR	1336 (55.7%)
EPMR	14 (0.6%)
ESD	4 (0.2%)
Histological diagnosis^‡^	
TA/TVA/VA with LGD	2,032 (85.7%)
TA/TVA/VA with HGD	31 (1.3%)
SSL	22 (0.9%)
Mucosal cancer	16 (0.7%)
Submucosal cancer^§^	10 (0.4%)
Others (HP and IP)	259 (10.9%)
Complication	
Delayed bleeding	6 (3.9%)
Perforation	0 (0.0%)

Data are presented as n (%) or mean ± standard deviation with median and range.

*CFP* cold forceps polypectomy, *CSP* cold snare polypectomy, *EMR* endoscopic mucosal resection, *EPMR* endoscopic piecemeal mucosal resection, *ESD* endoscopic submucosal dissection, *HGD* high-grade dysplasia, *LGD* low-grade dysplasia, *SSL* sessile serrated lesion, *TA* tubular adenoma, *TVA* tubulovillous adenoma, *VA* villous adenoma, *HP* hyperplastic polyp, *IP* inflammatory polyp.

*Colonoscopic polypectomy methods were analyzed in 2400 cases.

^‡^Histological diagnosis was analyzed in 2370 specimens retrieved after endoscopic resection.

^§^All submucosal cancers were superficial submucosal cancers with a submucosal invasion depth < 1000 µm from the muscularis mucosa without poor histological features (poorly differentiated adenocarcinoma, lymphovascular invasion, and tumor budding).

### Surveillance colonoscopy findings

The mean number of surveillance colonoscopies was 3.6 ± 1.8 (median 3, range 2–12) over a follow-up of 64.6 ± 30.1 months. Among patients with complete or near complete removal of all polyps at the baseline colonoscopy, the first surveillance colonoscopy interval was 13.2 ± 5.8 months. In contrast, it was 9.2 ± 5.9 months among patients with incomplete removal of all polyps at the baseline colonoscopy. The mean number of detected polyps at the first surveillance colonoscopy was 12.2 ± 23.4. Of these, 7.5 ± 7.4 polyps were endoscopically resected. The mean size of the largest polyp was 7.7 ± 4.3 mm. The procedure time ranged from 11 to 113 min (31.9 ± 18.9 min). The findings of the first surveillance colonoscopy are summarized in Table 3. Table 3 also shows detailed findings of the second and subsequent surveillance colonoscopies. The number and size of polyps decreased as surveillance colonoscopies were repeated compared to those at baseline colonoscopy (Table 3). In addition, the frequencies of CFP and CSP increased as surveillance endoscopy was repeated, whereas that of EMR decreased. Finally, the surveillance interval became longer as surveillance colonoscopies were repeated.

**Table 3:** Surveillance colonoscopy findings.

*The first surveillance colonoscopy*	
Number of polyps detected	12.2 ± 23.4 (median 5, range 1–200)
Number of polyps resected	7.5 ± 7.4 (median 5, range 1–49)
Size of the largest polyp, mm	7.7 ± 4.3 (median 7, range 1–30)
Colonoscopy procedure time, min	31.9 ± 18.9 (median 26.5, range 11–113)
< 30 min	91 (59.9%)
30–59 min	51 (33.5%)
60–89 min	7 (4.6%)
≥ 90 min	3 (2.0%)
Colonoscopic polypectomy method*	
CFP	446 (41.7%)
CSP	186 (17.4%)
EMR	433 (40.5%)
EPMR	5 (0.4%)
ESD	0 (0.0%)
*The second surveillance colonoscopy*	
Number of polyps detected	10.5 ± 23.1 (median 5, range 0–100)
Number of polyps resected	6.4 ± 6.9 (median 5, range 0–47)
Size of the largest polyp, mm	7.0 ± 4.7 (median 5.5, range 2–35)
Colonoscopy procedure time, min	26.5 ± 12.8 (median 23, range 9–85)
< 30 min	108 (71.0%)
30–59 min	41 (27.0%)
60–89 min	3 (2.0%)
≥ 90 min	0 (0.0%)
Colonoscopic polypectomy method	
CFP	490 (53.5%)
CSP	213 (23.2%)
EMR	210 (22.9%)
EPMR	3 (0.3%)
ESD	1 (0.1%)
*Subsequent surveillance colonoscopies*	
Number of polyps detected	11.6 ± 21.2 (median 5, range 0–100)
Number of polyps resected	7.1 ± 7.3 (median 5, range 0–67)
Size of the largest polyp, mm	6.7 ± 4.7 (median 5, range 2–28)
Colonoscopy procedure time, min	27.9 ± 14.6 (median 24, range 10–80)
< 30 min	149 (66.2%)
30–59 min	64 (28.5%)
60–89 min	12 (5.3%)
≥ 90 min	0 (0.0%)
Colonoscopic polypectomy method	
CFP	815 (56.9%)
CSP	373 (26.0%)
EMR	233 (16.3%)
EPMR	12 (0.8%)
ESD	0 (0.0%)

Data are presented as n (%) or mean ± standard deviation with median and range.

*CFP* cold forceps polypectomy, *CSP* cold snare polypectomy, *EMR* endoscopic mucosal resection, *EPMR* endoscopic piecemeal mucosal resection, *ESD* endoscopic submucosal dissection.

The cumulative incidence of metachronous advanced neoplasia including cancer, adenoma ≥ 1 cm, and adenoma with high-grade dysplasia and/or villous component, was 6.3% at 1 year, 11.9% at 3 years, and 15.6% at 5 years. Advanced cancer was not detected in any patients during the follow-up period. Early cancers were diagnosed during surveillance colonoscopies in three patients (Table 4). The intervals between prior colonoscopy and early cancer diagnosis were 6, 12, and 21 months, respectively. The size of each cancer was 7, 8, and 12 mm. All three early cancers were completely resected using EMR. Histological examination showed mucosal cancer with clear resection margins in all three cases. Cancer recurrence did not develop during the 18–55-month follow-up after endoscopic resection of early cancer in these patients.

**Table 4:** Summary of three patients diagnosed with early cancer during surveillance colonoscopies.

	Sex & age	Prior colonoscopy findings before diagnosis of early cancer during the surveillance colonoscopy				Interval from the previous colonoscopy	Findings of early cancer during the surveillance colonoscopy						
		Number of polyps	The largest polyp size	The most advanced histology	Complete removal of all polyps		Location of early cancer	Size of early cancer	Morphology of early cancer	Depth of invasion	Treatment	F/U after treatment	Cancer recurrence
Patient 1	M/64	4	10	TA with LGD	Yes	21 months	Transverse colon	12 mm	LST-NG-FE	Mucosa	EMR	55 months	No
Patient 2	M/54	12	16	TA with LGD	Yes	12 months	Rectum	8 mm	Paris type Is	Mucosa	EMR	32 months	No
Patient 3	M/49	26	20	TVA with HGD	No	6 months	Sigmoid colon	7 mm	Paris type Is	Mucosa	EMR	18 months	No

*EMR* endoscopic mucosal resection, *F/U* follow-up, *HGD* high-grade dysplasia, *LGD* low-grade dysplasia, *TA* tubular adenoma, *TVA* tubulovillous adenoma, *LST-NG-FE* laterally spreading tumor-non granular-flat elevated.

Delayed bleeding occurred in 2 (0.4%) of the 529 surveillance colonoscopies. All cases of delayed bleeding were successfully managed conservatively using endoscopic hemostasis. No perforation occurred during 529 surveillance colonoscopies.

Surgery was unnecessary for any patients because of detection of endoscopically incurable cancer or colonoscopy-associated adverse events.

## Discussion

In our large cohort of patients who underwent repeat colonoscopic polypectomy after resection of ≥ 10 colorectal polyps at the baseline colonoscopy, only 3 of 152 patients developed mucosal cancer. All three lesions were endoscopically resected. No cancer recurrence occurred after the endoscopic resection. Delayed bleeding occurred in 6 (3.9%) of the 152 baseline colonoscopies and in 2 (0.4%) of the 529 surveillance colonoscopies. All delayed bleedings episodes were successfully managed using endoscopic hemostasis. No perforation occurred. Surgery was not required for any reason, including adverse colonoscopy events or the occurrence of endoscopically incurable cancer.

The effectiveness of screening and surveillance colonoscopies can be assessed by achieving their goal, which is reducing colorectal cancer mortality and possible prevention of colorectal cancer by polypectomy^2,3^. Therefore, we investigated colorectal cancer development and mortality as surrogate markers of the effectiveness of surveillance colonoscopy with repeat polypectomy in patients with ≥ 10 polyps at baseline colonoscopy. In our analyses, colorectal cancer was diagnosed in 3 patients (2.0%) during surveillance colonoscopies. However, all cancers were confined to the mucosal layer (pTis) and could be treated by endoscopic resection without surgery or chemotherapy. No colorectal cancer-related mortality occurred during the follow-up period. Few studies have investigated the effectiveness of repeat polypectomy as a primary interest in patients with ≥ 10 polyps. In a previous study that investigated the usefulness of repeat polypectomy in 90 patients diagnosed with familial adenomatous polyposis, colorectal cancer occurred in 5 (5.6%) during the follow-up period. All the cancers were treatable via endoscopic resection. No recurrence or metastasis was observed^11^. Based on the results of that study and our experience, we suggest that repeat colonoscopic polypectomy can effectively prevent colorectal cancer and reduce mortality in patients with ≥ 10 polyps.

For widespread adoption of surveillance colonoscopy with repeat polypectomy in patients with ≥ 10 polyps in clinical practice, not only oncological effectiveness but also clinical efficiency should be secured. Therefore, we investigated procedure time, an indicator of time-effectiveness, as a surrogate marker of clinical efficiency. The mean procedure time of the baseline colonoscopy was 54.9 min, and only 10 (6.6%) were ≥ 90 min, which may be an acceptable range of procedure time in clinical practice. The mean procedure time of the first surveillance colonoscopy was 31.9 min, and a procedure time ≥ 90 min was required in only three (2.0%) patients. The procedure time of subsequent surveillance colonoscopies was shorter. These findings suggest that the time-effectiveness improves for surveillance colonoscopies because of the smaller number and size of remaining/recurrent polyps after initial polypectomy primarily for large polyps at the baseline colonoscopy. An interesting point related to procedure time was the resection method. EMR was the most frequently performed resection method during the baseline colonoscopy, accounting for 55.7% of all polypectomies performed. CSP was performed in only 17.1% of polypectomies; however, this study included patients treated beginning in 2004, when CSP was not widely performed. Because of its safety and high complete resection rate^12–14^, CSP is currently the most commonly used polypectomy method for diminutive and small polyps. Considering its technical simplicity and shorter procedure time compared to EMR, the time-effectiveness of repeat colonoscopy management of patients with ≥ 10 polyps may be even better if CSP is more commonly used. Finally, the proportion of EMR performed decreased with an increasing number of subsequent surveillance colonoscopies. In contrast, the proportions of CSP and CFP increased, suggesting good overall time effectiveness of the strategy of repeat colonoscopy management.

The post-polypectomy bleeding rate was 0.44% in a previous analysis of 15,285 colonoscopies^8^, and a meta-analysis analyzing 1,966,340 colonoscopies in 21 studies found a post-polypectomy bleeding rate of 0.98% and a perforation rate of 0.08%^10^. In our study, although perforation did not occur, the delayed bleeding incidence was 3.9% after polypectomy of ≥ 10 polyps at the baseline colonoscopy, which is higher than the 0.44–0.98% reported in previous studies on conventional colonoscopic polypectomy^8,10^. However, the frequency of clinically significant bleeding after wide-field EMR for large polyps (≥ 20 mm) was 6.7% in a multicenter study^15^. Other studies analyzing the outcomes of EPMR showed post-polypectomy bleeding rates of 2.3–8.8%^16–18^. Considering these bleeding frequencies, the delayed bleeding rate of 3.9% after polypectomy of ≥ 10 polyps may be clinically acceptable. In addition, delayed bleeding occurred in only 0.4% of surveillance colonoscopies. Finally, all bleeding episodes were successfully treated without surgery. These findings suggest that repeat colonoscopy may be safe for patients with ≥ 10 polyps.

The cumulative incidence of metachronous advanced neoplasia is an important factor when recommending surveillance colonoscopy intervals. Previous studies including general populations with no focus on patients having ≥ 10 polyps showed that the cumulative incidences of metachronous advanced neoplasia were 3.9–4.7% at 3 years and 4.9–6.3% at 5 years after the removal of 1–2 low-risk adenomas. They were 5.9–6.8% at 3 years and 10–12.2% at 5 years after removal of ≥ 3 low-risk adenomas^19–22^. The cumulative incidence of metachronous advanced neoplasia at 1 year was 6.3% in our study, which corresponds to an incidence of 5.9–6.8%, the 3-year incidence after removal of ≥ 3 low-risk adenomas in previous studies, for which 3-year surveillance colonoscopy was traditionally recommended. Nonetheless, we suggest a 1-year surveillance colonoscopy after complete or near-complete removal of ≥ 10 polyps would be adequate because multiple polyps at baseline colonoscopy were a risk factor for missed adenomas, an important cause of interval cancer^23,24^. Therefore, we performed surveillance colonoscopies at 1-year intervals if all polyps were benign and removed at baseline colonoscopy, as recommended by the USMSTF 2020 recommendation^25^. However, if some low-risk polyps remained in situ after removal of high-risk polyps at baseline colonoscopy, surveillance colonoscopy was performed 6–9 months later to clear the remaining polyps. If malignant polyps were removed at baseline colonoscopy, the first surveillance colonoscopy was performed within 6 months, which is similar to the USMSTF 2016 recommendation of a 3–6-month interval for endoscopy after endoscopic resection of early rectal cancer^26^. Using this surveillance strategy, we could minimize colorectal cancer development and eliminate colorectal cancer mortality effectively, efficiently, and safely. Therefore, based on the results of our study and previous guidelines, we suggest a 1-year interval for surveillance colonoscopy after removal of ≥ 10 polyps at baseline colonoscopy. Earlier surveillance is recommended if a considerable number of polyps remain or if early cancers are resected. Modification of the subsequent surveillance intervals should be made based on the number of polyps in the first and second surveillance colonoscopies. Usually, a gradual increase in the interval for subsequent surveillance colonoscopies can be recommended based on the decreasing polyp burden because of the prior polypectomy.

This study has several limitations. First, cost-effectiveness analysis was not performed. Because patients with ≥ 10 polyps require long-term follow-up, the cost-effectiveness of repeat colonoscopies should be compared to that of other management options, such as surgery, thereby identifying the best management method from all clinical viewpoints. Second, genetic tests were conducted in only a minority of included patients. Therefore, it was difficult to analyze the usefulness of a repeat colonoscopy strategy along with other surveillance tests for other high-risk organs, such as the stomach, duodenum, and thyroid, where extra-colonic malignancies can develop in patients with genetically confirmed hereditary polyposis syndromes. Third, this study was a retrospective analysis, and the surveillance intervals were not completely consistent across the cohort. Finally, small number (mean 3.6) of surveillance colonoscopies over only 5 years in 152 patients in a single institution may make a confirmative conclusion difficult in this study. Considering the necessity of lifelong management of metachronous polyps, further prospective, large scale, long-term studies adopting a strict surveillance interval strategy are needed for more confirmative analysis.

In conclusion, colonoscopic polypectomy with repeat surveillance colonoscopies is a clinically effective, efficient, and safe management option in patients with ≥ 10 polyps. Repeat colonoscopy can minimize the risk of colorectal cancer development and mortality in these patients.

## Methods

### Study design

**Figure 2:**
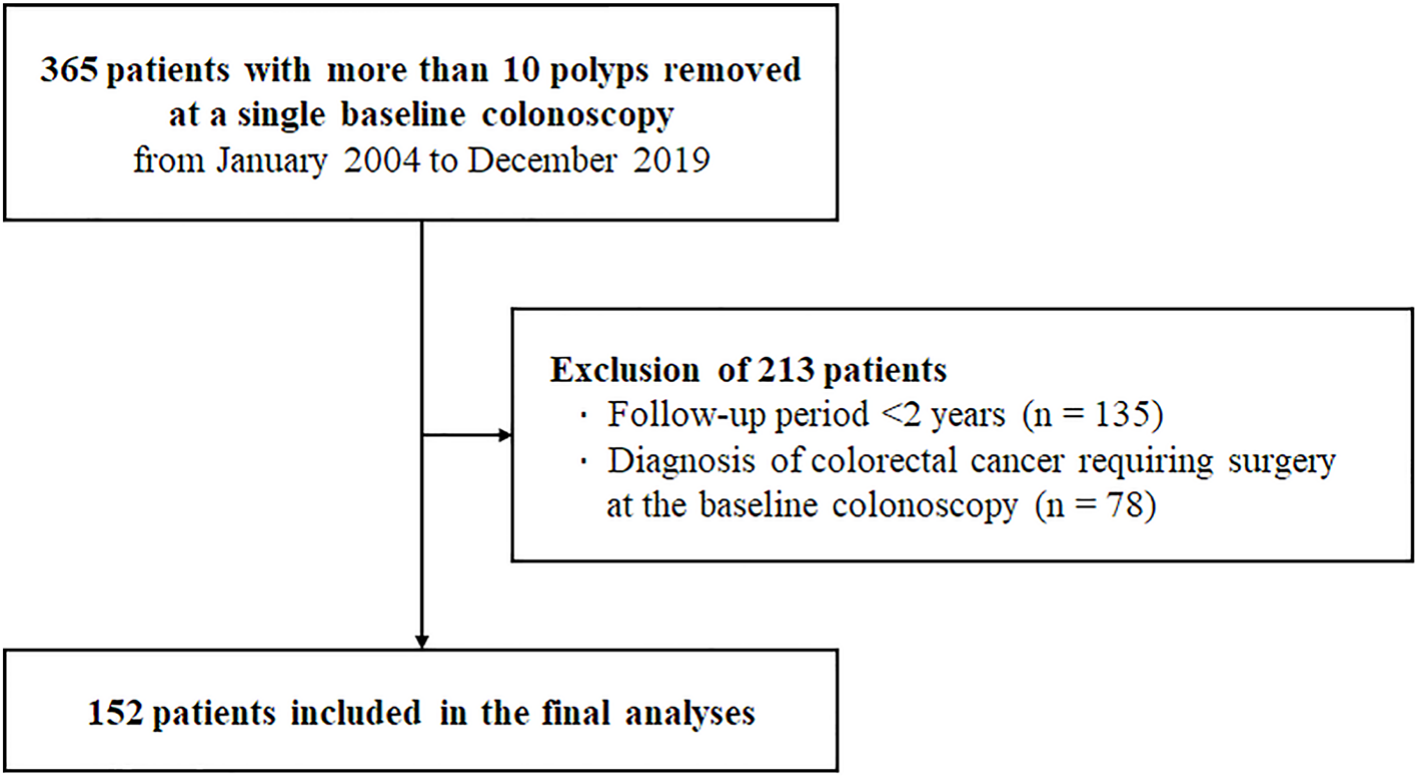
Flow chart for inclusion of patients.

This study was a retrospective review of the medical records of patients in whom multiple colorectal polyps (≥ 10) were removed at a baseline colonoscopy at the Asan Medical Center, Seoul, from January 2004 to December 2019. A review of the colonoscopy and histology database of our institution initially identified 365 patients with ≥ 10 polyps removed in a single colonoscopy. Of these, 213 patients were excluded because of a follow-up period of < 2 years or a diagnosis of colorectal cancer requiring surgery at baseline colonoscopy (Fig. 2). Therefore, 152 patients were included in the final analyses. Medical records, including colonoscopy reports, were also reviewed. Age, sex, and family history of colorectal cancer were also assessed. If genetic tests for hereditary polyposis syndrome were performed, the results were also investigated. The numbers of polyps detected and removed at the baseline and surveillance colonoscopies were assessed. The number of polyps was described as precisely as possible; however, when more than approximately 50 polyps were observed, the number of polyps was described roughly in units of 10. The size of detected polyps and histology of resected polyps at baseline and surveillance colonoscopies were also investigated, as were the total number of surveillance colonoscopies and duration of follow-up. The protocol of this study was approved by the Asan Medical Center Institutional Review Board (IRB 2020–1696). Written informed consent was not obtained from participants because of the retrospective study design. The institutional review board of our institution waived the need for informed consent based on the non-invasive and anonymized nature of this study. This study was performed in accordance with institutional ethical guidelines and the Declaration of Helsinki.

### Colonoscopic polypectomy at the baseline colonoscopy

**Figure 3:**
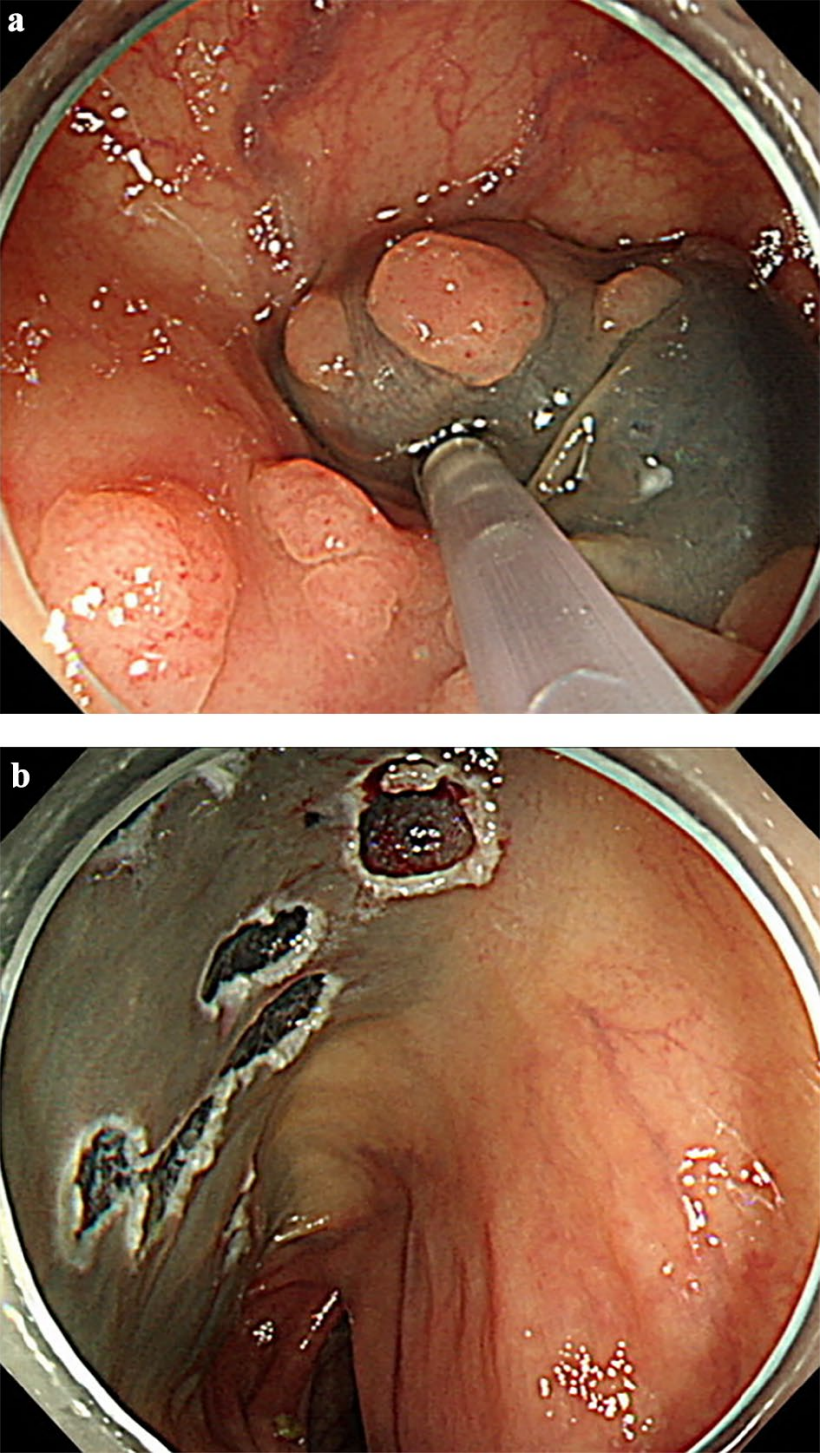
Colonoscopic polypectomy of multiple polyps. (**a**) Endoscopic mucosal resection (EMR) was performed for multiple polyps. (**b**) Multiple post-EMR ulcers were noted.

The general principles of colonoscopic polypectomy at baseline colonoscopy are as follows. First, complete resection of all polyps was attempted if the number of polyps was modest (approximately ≤ 20). Second, if the number of polyps was high, high-risk polyps were removed, and some low-risk polyps were left in situ. High-risk polyps included polyps ≥ 10 mm in size and those with surface features suggesting high-grade dysplasia or cancer based on pit pattern analysis and narrow band imaging analysis. Third, all the resected polyps were retrieved for histological examination. However, diminutive polyps (< 5 mm) assessed as low-risk with high confidence could be discarded after endoscopic resection. The colonoscopy techniques used in this study included CFP, CSP, EMR, EPMR, and ESD (Fig. 3).

Colonoscopy procedure time and adverse events such as delayed bleeding and perforation were reviewed. Colonoscopy procedure time was defined as the time from the insertion of the colonoscope through the anus to withdrawal of the scope. Delayed bleeding was defined as hematochezia or melena that required endoscopic hemostasis after completion of colonoscopy. The perforation was diagnosed endoscopically or radiologically.

### Surveillance colonoscopy

The interval between the baseline colonoscopy and the first surveillance endoscopy was determined based on the size, number, and histology of the polyps and completeness of endoscopic resection at the baseline colonoscopy. In general, surveillance colonoscopy was performed after 1 year if all polyps were removed and were benign. If some low-risk polyps remained in situ after removal of high-risk polyps at the baseline colonoscopy, surveillance colonoscopy was performed after 6–9 months. If malignant polyps were removed at the baseline colonoscopy, the first surveillance colonoscopy was performed within 6 months. The principles of colonoscopic polypectomy of the surveillance colonoscopy are similar to those of baseline colonoscopy.

The intervals between the first and subsequent surveillance colonoscopies were decided according to the findings of the first surveillance colonoscopy. If ≤ 3 polyps < 10 mm were removed, the next surveillance colonoscopy was performed at 3 years. If ≥ 10 polyps were removed, the next surveillance colonoscopy was performed at 1 year. If 4–9 polyps were removed, the next surveillance colonoscopy was performed between 1 and 3 years at the discretion of the endoscopist. Surveillance colonoscopy intervals can be modified if clinically indicated. The surveillance colonoscopy procedure time and adverse events were also reviewed.

### Statistical analysis

Categorical and nominal variables are expressed as numbers with percentages, and continuous variables are presented as means ± standard deviations. Analysis of variance, Student’s t-test, and chi-squared test were performed to examine differences among groups. Statistical analyses were performed using Microsoft Office Excel 2010 (Microsoft, Redmond, WA, USA) and IBM SPSS Statistics for Windows, version 24.0 (IBM Corp., Armonk, NY, USA).

## Data Availability

The datasets generated and/or analysed during the current study are not publicly available due to privacy of patients. When this study approved by IRB, data should be discarded without taking it out after use but datasets are available from the corresponding author on reasonable request.
